# Separation and identification of celery proteins

**DOI:** 10.1186/2045-7022-4-S2-P55

**Published:** 2014-03-17

**Authors:** Serena O'Neil, Katrin Darm, Laura Rethschulte, Christian Scharf

**Affiliations:** 1 University of Medicine Greifswald, ENT Research Laboratory, Greifswald, Germany

## Background

Allergy to Apium graveolens is one of the more severe food allergies. Six celery allergens have been characterised, Api g 1-6, while additional allergens have characterised from other members of the Magnoliospida class, including kiwi, peanut and ragweed, suggesting homologous allergens may also be present in celery. The participants of the Omics Allergy School 2013 were provided with celery bulb and leaf extracts for two dimensional separation and immunoblotting.

## Methods

Two dimensional electrophoresis was used to separate the bulb and stalk protein extracts of two strains of celery. Allergenic proteins were detected using sera from a celery allergic patient.

## Results

Hundreds of celery bulb and stalk proteins were separated using 2DE, using both silver stain and fluorescent labelling for increased sensitivity. The protein profile of two celery strains were distinctly different. A similar IgE binding pattern was observed to the bulb of the two strains of celery with 20-30 spots detected, while the binding pattern of the leaves of the two strains differed. Proteins have been identified by MALDI-ToF-MS/MS, with a de novo-sequencing approach used for protein identification due to limited protein sequences for celery. Post translational modifications of protein isoforms have been quantitatively labelled by H216O/ H218O-trypsin and subsequently analysed by nanoLC-ESI-MS/MS. PNGase treatment has been used for addressing protein glycosylation.

## Conclusion

Allergy School participants were able to contribute to the separation of celery extracts and to the identification of celery allergens.

